# Using Internet-Based Psychological Measurement to Capture the Deteriorating Community Mental Health Profile During COVID-19: Observational Study

**DOI:** 10.2196/20696

**Published:** 2020-06-11

**Authors:** Joep van Agteren, Jonathan Bartholomaeus, Daniel B Fassnacht, Matthew Iasiello, Kathina Ali, Laura Lo, Michael Kyrios

**Affiliations:** 1 Wellbeing and Resilience Centre Lifelong Health Theme South Australian Health and Medical Research Institute Adelaide Australia; 2 College of Education, Psychology and Social Work Flinders University Adelaide Australia; 3 Órama Institute for Mental Health and Wellbeing Adelaide Australia; 4 School of Psychology The University of Adelaide Adelaide Australia; 5 College of Nursing and Health Science Flinders University Adelaide Australia

**Keywords:** psychological measurement, positive mental health, resilience, mental health, psychological distress, internet, COVID-19, pandemic

## Abstract

**Background:**

The coronavirus disease (COVID-19) is expected to have widespread and pervasive implications for mental health in terms of deteriorating outcomes and increased health service use, leading to calls for empirical research on mental health during the pandemic. Internet-based psychological measurement can play an important role in collecting imperative data, assisting to guide evidence-based decision making in practice and policy, and subsequently facilitating immediate reporting of measurement results to participants.

**Objective:**

The aim of this study is to use an internet-based mental health measurement platform to compare the mental health profile of community members during COVID-19 with community members assessed before the pandemic.

**Methods:**

This study uses an internet-based self-assessment tool to collect data on psychological distress, mental well-being, and resilience in community cohorts during (n=673) and prior to the pandemic (two cohorts, n=1264 and n=340).

**Results:**

Our findings demonstrate significantly worse outcomes on all mental health measures for participants measured during COVID-19 compared to those measured before (*P*<.001 for all outcomes, effect sizes ranging between Cohen d=0.32 to Cohen d=0.81. Participants who demonstrated problematic scores for at least one of the mental health outcomes increased from 58% (n=197/340) before COVID-19 to 79% (n=532/673) during COVID-19, leading to only 21% (n=141) of measured participants displaying good mental health during the pandemic.

**Conclusions:**

The results clearly demonstrate deterioration in mental health outcomes during COVID-19. Although further research is needed, our findings support the serious mental health implications of the pandemic and highlight the utility of internet-based data collection tools in providing evidence to innovate and strengthen practice and policy during and after the pandemic.

## Introduction

### Background

The advent of the coronavirus disease (COVID-19) and the widespread (social) control mechanisms implemented around the world are expected to lead to significant deterioration in mental health in the wider community; however, the magnitude of the damage is unknown [1,2]. The impact of the pandemic on health systems, the worsening economy and associated high rates of unemployment, the widespread social restrictions and quarantining, and the constant presentation of confronting news messages from the media are unprecedented challenges for communities and the mental health of its members [3-6].

It is crucial to thoroughly assess the potential community mental health consequences of the pandemic and gain insight into local data on mental health outcomes, as a shift in the mental health profile of the community will have short- and long-term consequences for health services, policy makers, and society in general. In addition to their ability to improve the psychological assessment process in general [7-9], internet-based measurement of mental health outcomes can play an important role in gathering data to inform policy and practice during and after the pandemic [10]. Such measurements inherently possess the ability to collect data on a large scale and facilitate immediate reporting on user mental health status, which ultimately can enhance participant mental health literacy and stimulate help-seeking behavior [11].

This is particularly relevant in light of the reduced ability and opportunity to conduct traditional assessments and screening for mental illness during COVID-19 as a result of physical distancing protocols. There is an important role to play for internet-based measurement of the general distress and well-being profile of the community, which is more suitable for online testing compared to assessment of specific disorders or severe mental illness. Higher rates of community distress and significant deterioration of positive and adaptive states of mental health—mental wellbeing and resilience—can signal the immediate and long-term presence of mental illness in the wider population [12-14]. As such, they are key indicators of the deterioration of mental health in the general community.

### The Australian Context

As of the June 2, 2020, Australia had a total of 7204 confirmed cases of COVID-19, resulting in 103 deaths (1.4% death rate) [15]. The first reported case of COVID-19 in Australia was on January 25, 2020, in Victoria [16]. The Australian Government Department of Health [17] reported that cases peaked in March, and since April, the number of identified cases have remained relatively low. Of the states and territories within Australia, on June 2, 2020, New South Wales had the highest number of COVID-19 cases (3104 cases), followed by Victoria (1663 cases), Queensland (1059 cases), Western Australia (591 cases), South Australia (440 cases), Tasmania (228 cases), Australian Capital Territory (107 cases), and Northern Territory (29 cases) [18]. To put these numbers in perspective, rates in Australia are approximately 282 per 1 million compared with 5184 in the United States, 4009 in the United Kingdom, and 3848 in Italy [19].

In an attempt to flatten the curve of COVID-19 in Australia, the Australian Government began to slowly introduce lockdown measures. On February 1, 2020, travelers from mainland China were required to self-isolate for a period of 14 days from the date they left China, but widespread societal measures were not considered. Early March 2020 saw the rise of panic buying in supermarkets, and, through the rest of the month, the federal and state governments established progressively tighter lockdown restrictions, including limiting social gatherings and nonessential travel, and lockdowns of gyms, bars, restaurants, and schools [20]. Other nonessential workplaces instructed their staff to work from home where possible. With the exception of minor incidents, the rules on restrictions were generally followed by the Australian population, despite its flow-on effects resulting in many thousands of people losing their livelihoods, as well as resulting in large-scale social change and restrictions of freedom.

### This Study

The impact of the COVID-19 pandemic in Australia has had a serious impact on a community and societal level. As a result, it could be expected to negatively impact mental health outcomes in the wider community, not simply limited to those directly affected or exposed to the illness. Therefore, it is important to quantify the local impact of the pandemic on community mental health outcomes, data that can feasibly be gathered using internet-based tools and methods. This study investigates the mental health outcomes of Australian community members accessing internet-based mental health assessment and psychological skills training during COVID-19 in comparison to cohorts of people engaged in these services prior to the pandemic.

## Methods

### Participants and Recruitment

Participants were adults who engaged with services offered by the South Australian Health and Medical Research Institute (SAHMRI) Wellbeing and Resilience Centre, based in Adelaide, Australia. The center provides internet-based measurement of mental health and well-being, and delivers general psychological skills training to the general community, with the aim of improving mental health and well-being.

Participants recruited during COVID-19 (hereafter, COVID-19 group) registered to participate in the study from March 29, 2020, onwards, well into the period of social restrictions in Australia, which occurred mid-March. Recruitment was conducted via two weblinks. The first was from the generic website of the Wellbeing and Resilience Centre, where people could find mental health resources and register for a free evidence-based mental health and well-being measurement. The second is a website that provides information about free online psychological skills training called the Be Well Plan, where participants could preregister and complete the same mental health and well-being measurement offered on the first website, prior to commencing the training.

Participants from the comparator cohort were adults who participated in mental health and well-being measurement and training between February 2019 and February 14, 2020, a month before social restrictions were implemented and when incidence of COVID-19 in Australia remained low. The first comparator cohort (general [GEN] group) consisted of participants who either took part in a group-based psychological skills training that was offered prior to COVID-19 or registered via the generic SAHMRI website for a free well-being measurement. Participants for the training included individuals from the public or people recruited for specific projects, for example, training provision to workforces. Participants from those projects were not community members reaching out on their own accord (ie, their employer may have directed them to participate), which meant their motivation could have been different to the COVID-19 cohort. This led to the creation of a second comparator cohort (help-seeking [HELP] group), consisting of individuals from the general population who engaged in the training or measurement of their own volition.

After registration, all participants completed the measurement online via internet-enabled devices (approximately 10-15 minutes to complete). The measurement captured basic demographic information (ie, gender, age, employment, and study status) to keep questionnaire burden low. After completing the measurement, participants were automatically provided with their individual scores and an individualized online report that explained the results and provided information about subsequent options to improve their mental health, as well as information on mental health services in case of immediate need.

### Outcome Measures

The measurement included items assessing psychological distress associated with symptoms of depression and anxiety, as well as positive (mental well-being) and adaptive (resilience) states. Psychological distress was measured using the Depression Anxiety and Stress Scale-21 items (DASS-21) [21]. The DASS-21 offers reliable cut-off points for symptom severity (ie, “mild,” “moderate,” “severe,” and “extremely severe” symptoms). Analyses were conducted using total scores for each of the three domains; internal consistencies for depression (α=.92), anxiety (α=.84), and stress (α=.86) were good. Well-being was measured using the Mental Health Continuum Short-Form (MHC-SF) [22]. The MHC-SF is a valid and reliable measure of mental well-being, providing both a continuous measure of three key domains of well-being (hedonic, eudaimonic, and social well-being), as well as a “diagnosis” of overall well-being into “flourishing” or high well-being, moderate well-being, and “languishing” or low well-being. Internal consistency was assessed on the summed total score of all 14 items (α=.94). An additional well-being measure was used to specifically capture satisfaction with life. The Satisfaction With Life Scale [23] is a universally accepted measure, demonstrating high internal consistency (α=.91). Adaptive states were measured using the Brief Resilience Scale (BRS) [24]. The BRS conceptualizes resilience as an outcome and is a well-accepted tool to gain insight into resilience, with cut-offs for low, normal, and high resilience. Internal consistency was high, α=.88.

### Data Analysis

Independent samples *t* tests and chi-square tests were conducted to investigate demographic differences between groups. Differences between groups were assessed using multivariate analysis of variance to test for an overall difference between conditions and subsequent analyses of variance to test for differences in each dependent variable. Covariates were entered to control for any baseline differences between the groups in the analyses. Given that all dependent variables were moderately correlated, a Bonferroni correction for multiple comparisons was employed, using an alpha level of =.008. Listwise deletion was employed to handle missing data. The previously mentioned measurement cut-offs were used to determine whether participants were “healthy” compared to participants who demonstrated distress or at-risk scores; healthy participants referred to high levels of well-being, normal levels of resilience, and no symptoms of distress in any of the three domains.

## Results

The COVID-19 group consisted of 673 participants, while the control cohorts consisted of 1264 participants and 340 participants from the GEN group and HELP group, respectively. Demographic characteristics of the cohorts are reported in Table 1. There were less males in the COVID-19 sample compared to the two control cohorts (χ^2^_2_=194.1, *P*<.001), and the average age in the COVID-19 cohort was marginally higher (*F*_2,2274_=3.56, *P*=.03, *η*^2^_partial_=0.003). A significant difference also existed in the proportion of participants employed (χ^2^_2_=243.1, *P*<.001), as the COVID-19 cohort consisted of more unemployed participants. Finally, there were significantly less people studying in the GEN group compared to the other two cohorts (χ^2^_2_=243.1, *P*<.001) in each sample. As a result, age, gender, study, and employment status were controlled for in the subsequent analyses.

**Table 1:** Demographics.

Demographic	COVID-19^a^ (n=673)^b^	GEN^c^ (n=1264)^d^	HELP^e^ (n=340)^f^
Age (years), mean (SD)	44.8 (14.7)	42.7 (11.4)	42.6 (11.8)
Gender (female), n (%)	437 (65)	583 (46)	198 (58)
Unemployed, n (%)	168 (25)	30 (2)	36 (11)
Studying, n (%)	107 (16)	46 (4)	53 (16)

^a^COVID-19: coronavirus disease.

^b^The COVID-19 cohort consists of participants recruited in March and April 2020.

^c^GEN: general.

^d^The GEN cohort consists of participants engaging in mental health training and measurement during February 2019 to February 2020.

^e^HELP: help-seeking.

^f^The HELP cohort is a subset of the general cohort, which consists of users who reached out to the service on their own accord (as opposed to being invited as part of a specific project).

There was a significant multivariate difference between the three samples on all outcome measures (Pillai *V*=0.17, *F*_2,2268_=35.66, *P*<.001, *η*^2^_partial_=0.06; refer to Table 2 for means and SDs for all outcome variables). Subsequent univariate analyses indicated a significant difference between the cohorts on depression (*F*_2,2268_=93.8, *P*<.001, *η*^2^_partial_=0.051), stress (*F*_2,2268_=47.8, *P*<.001, *η*^2^_partial_=0.066), anxiety (*F*_2,2268_=108.8, *P*<.001, *η*^2^_partial_=0.031), well-being (*F*_2,2268_=28.8, *P*<.001, *η*^2^_partial_=0.017), life satisfaction (*F*_2,2268_=44.2, *P*<.001, *η*^2^_partial_=0.020), and resilience (*F*_2,2268_=150.5, *P*<.001, *η*^2^_partial_=0.075). Tukey post-hoc comparisons indicated that the COVID-19 cohort showed significantly worse outcomes compared to both control cohorts on depression, stress, anxiety, well-being, life satisfaction, and resilience (Table 2). No differences between the two control cohorts were found for general well-being, life satisfaction, depression, and anxiety. The GEN group differed significantly from the HELP group in stress and resilience, with the HELP group showing worse outcomes.

**Table 2:** Means and SDs for the COVID-19 and control cohorts.

Variables	COVID-19^a,b^, mean (SD)	GEN^c,d^, mean (SD)	HELP^e,f^, mean (SD)	COVID-19 vs GEN		COVID-19 vs HELP			GEN vs HELP	
				Cohen *d*^g^	*P* value	Cohen *d*	*P* value	Cohen *d*		*P* value
Depression	12.69 (10.56)	6.79 (8.52)	8.05 (8.63)	0.62	<.001	0.48	<.001	0.15		.06
Stress	16.11 (9.48)	10.14 (8.29)	12.48 (8.66)	0.67	<.001	0.40	<.001	0.28		<.001
Anxiety	8.41 (8.01)	5.22 (6.60)	5.84 (6.67)	0.44	<.001	0.35	<.001	0.09		.30
Well-being	42.87 (14.30)	47.35 (12.98)	47.85 (12.59)	0.33	<.001	0.370	<.001	0.04		.81
Life satisfaction	20.86 (6.98)	23.80 (6.55)	22.93 (6.11)	0.43	<.001	0.32	<.001	0.14		.08
Resilience	3.13 (0.81)	3.82 (0.90)	3.40 (0.81)	0.81	<.001	0.33	<.001	0.49		<.001

^a^COVID-19: coronavirus disease.

^b^The COVID-19 group consists of participants recruited in March and April 2020.

^c^GEN: general.

^d^The GEN group consists of participants who engaged in mental health training and measurement during February 2019 to February 2020.

^e^HELP: help-seeking.

^f^The HELP group is a subset of the general cohort, which consists of users reaching out on their own accord.

^g^Effect sizes were calculated using Cohen *d,* where 0.2 is a small effect, 0.5 is a medium effect, and 0.8 is a large effect.

Finally, the study investigated the proportion of participants that displayed problematic scores on at least one of the outcomes (ie, the proportion of participants with mental health problems). The COVID-19 cohort displayed a significantly higher proportion (n=532/673, 79%) of participants reporting problematic mental health outcomes, compared to the GEN (n=657/1264, 52%; χ^2^_2_=135.78, *P*<.001) and HELP cohort (n=197/340, 58%; χ^2^_2_=49.88, *P*<.001).

## Discussion

Our findings suggest a significant deterioration of mental health profiles for general community members engaging with mental health services during COVID-19 compared to before the global pandemic. All indicators of psychological distress as well as indicators of mental well-being and resilience were significantly lower, providing evidence to indicate the pervasive short-term mental health impact of the pandemic and the heightened risk of mental illness onset in the future for the general community [1,25].

The psychological impact of the COVID-19 pandemic observed in this study aligns with emerging global research and research into previous pandemics and disasters—research that largely focuses on distress and mental illness. Rajkumar [26] conducted a review of the literature related to mental health and the COVID-19 pandemic, indicating that elevated levels of anxiety, depression, and stress were the most common psychological reactions to COVID-19. Although this study did not investigate the exact mechanisms underpinning the psychological distress, the review also identified editorials and commentaries describing the potential mental health impacts of the pandemic, drawing from previous disease outbreaks. Unpredictability, uncertainty, severity of the diseases, social isolation and loneliness, misinformation, and economic impacts were cited among the factors most likely associated with the increased psychological distress [27,28]. Similar results on psychological distress were found in the context of the Korean Middles East respiratory syndrome outbreak [29], in medical staff following the Ebola outbreak in African nations [30], and in the severe acute respiratory syndrome–related coronavirus outbreak in Taiwan [31].

The impact of pandemics or lockdowns on positive and adaptive mental health states such as mental well-being has been researched far less than the impact on psychological distress; however, several determinants of mental well-being are impeded during the lockdowns. The clearest impact of the lockdown is on personal agency and autonomy, key determinants of psychological well-being and self-determination theory [32,33]. Recent research has validated the importance of loss in agency, showing that it may have a significant impact on levels of life satisfaction [34]. Physical activity is another strong determinant of mental well-being and distress, and a protective factor against psychological distress, which has been impacted by COVID-19–related restrictions [35]. The closure of gyms, sporting clubs, public parks, and recreational areas may have contributed to the results observed in this study [36]. Other important drivers of well-being [37] that were affected, and therefore can play a role in explaining the results found here, include spirituality and interpersonal relationships—as a result from places of worship, restaurants, bars, and universities closing—the loss of purpose or meaning in life due to financial distress or loss of employment, significant changes to lifestyle such as homeschooling children, and social isolation and loneliness to name a few.

The significant levels of distress in the current cohorts of Australian community members are alarming. First, they flag a deterioration of mental health profiles among the general nonclinical population, suggesting an urgent need for prevention or early intervention to improve mental health and well-being, and equip people with resources to better cope in times of adversity [25]. Mental well-being is a known protective factor from psychological distress and mental illness [14,38]; therefore, the deterioration in mental well-being is a cause for concern for the mental health in the mid- and long-term of the pandemic. Second, it is likely that levels of distress among people with mental disorders are even higher, pointing to an urgent need for local research and subsequently intervention when this is confirmed.

The HELP cohort was, on average, less resilient and experienced higher levels of stress compared to the GEN control cohort. The HELP cohort was constructed to lower the impact of bias between the COVID-19 group and the control group, as both the HELP and the COVID-19 cohorts proactively engaged with the services on their own volition. The results found in the study however make intuitive sense. Specifically, it can be expected that individuals suffering from increased levels of stress in their lives will seek out mental health service offerings to alleviate their stress. We further expect to see individuals low in self-reported levels of resilience (the measure of resilience used here focuses on feeling resilient to cope with stressful times [24]) to seek out the program to increase their self-perceived resilience. Taking these findings into account further highlights the impact of COVID-19 on participants mental health, as the COVID-19 cohort significantly deviated on all outcomes from the HELP cohort.

The results need to be placed in the Australian context, where the impact of COVID-19 has been less severe compared to countries in Europe or the United States and social control has been less restrictive. Furthermore, although the participants in this sample were seeking out mental health services, they most likely largely represented a nonclinical community sample. Significant levels of psychological distress were observed in participants, which may indicate the presence of mental disorders in some of the participants; the current findings should, however, not simply be generalized to demonstrate the impact of COVID-19 on people with (severe) mental disorders [39].

A silver lining to these results can be found, as the pandemic has triggered significant interest in mental health, and signs of acceleration and innovation in the way we measure and support mental health can already be seen; for example, increased access to electronic health apps for mental health [40,41]. The measurement used in this study was freely accessible via internet-enabled devices and resulted in an immediate, individualized report for the participants. Improving access to internet-based services can act as an important complement to face-to-face measurement methods, as it may reduce barriers to seeking help [42]. Harnessing internet-based innovations in mental health service provision can stimulate wider mental health reform and help strengthen services for the entire population, regardless of the presence of (severe) mental disorders [43]. This is particularly important in relation to access to mental health services for vulnerable groups. The results here, for instance, suggest that unemployed people are reaching out for help with their mental health, which, in light of the mass unemployment recorded around the world, has important implications for mental health care services resourcing across the spectrum [44].

A number of areas, highlighted by our findings, will provide fruitful avenues for future research. First, although we have shown that COVID-19 had a detrimental impact on participant’s mental health in general, understanding specifically the effects that COVID-19 may exert on people already experiencing a mental illness is important [1]. This pandemic may have compounded the issues already faced by a proportion of the population, particularly those who are the most vulnerable; understanding this interaction is the first step to providing more effective help to at-risk and mentally ill people in the community while facing adversity. Second, our findings suggest that the COVID-19 pandemic, as a whole, was detrimental to individuals’ mental health. However, our findings were unable to disentangle the specific mechanisms of decreased mental health during this pandemic. For instance, was social isolation the primary driver of decreased mental health, was it the loss of economic certainty, or was it caused more so by fear induced by media reporting? Future in-depth research of which determinants underpin the mental health impact of pandemics is required (eg, the role of social determinants of health [45]). Third, research and intervention aimed to improve psychological resilience may prove an economical way to improve community coping with large scale negative events. Future research on the mental skills that foster psychological resilience will enable the promotion of positive mental health in general and during widespread negative events [46], such as a pandemic, thus reducing the negative psychological impact of these events on the community.

In summary, more research is needed, particularly in monitoring the long-term consequences and determining the clinical impact of COVID-19 in different populations. This study is limited in its use of generic outcomes and its cross-sectional design. This means that more rigorously controlled studies are essential to capture the complexity of mental health amid a global pandemic. Our findings, however, demonstrate the utility of internet-based psychological measurement and contribute valuable data to equip stakeholders with evidence to further understand the considerable negative consequences of the COVID-19 pandemic—results that can be used to intervene and prevent amplification of its impact on community mental health.

## Abbreviations

BRS: Brief Resilience Scale
COVID-19: coronavirus disease
DASS-21: Depression Anxiety and Stress Scale-21 items
GEN: general
HELP: help-seeking
MHC-SF: Mental Health Continuum Short-Form
SAHMRI: South Australian Health and Medical Research Institute
